# Investigating the socioeconomic impacts of sewage spillages on businesses in the Umhlanga Rocks coastline area

**DOI:** 10.4102/jamba.v16i1.1602

**Published:** 2024-04-09

**Authors:** Kalin Naidoo, Ntombifuthi P. Nzimande, Feroza Morris

**Affiliations:** 1 Department of Geography, Faculty of Humanities, University of KwaZulu-Natal, Durban, South Africa; 2 Department of Geography, Faculty of Science, University of KwaZulu-Natal, Durban, South Africa

**Keywords:** sewage spillages, eThekwini municipality, business resilience, environmental pollution, socioeconomic impact, Umhlanga Rocks coastline

## Abstract

Sewage spillage is fast becoming a significant concern in South Africa because of the deteriorating infrastructure and substandard service delivery. Thus, this study investigated the socioeconomic impacts of sewage spillages on businesses in the Umhlanga Rocks coastline area. A questionnaire with open and closed-ended questions was used to collect data from business owners in the affected area. One hundred business owners were handed questionnaires to complete, and 95 completed questionnaires were collected. The qualitative data from the questionnaires were analysed using thematic analysis, whereby line-by-line coding was manually completed using Excel. The quantitative data were analysed in R studio using numerous statistical tests, including the Shapiro–Wilk test, the Chi–square test, the Kruskal–Wallis test, Dunn’s test, and Cohen’s d test. The thematic analysis and statistical testing results showed that sewage spillages disrupted businesses significantly. Results indicated that businesses of different sizes experienced socioeconomic losses differently. Consequently, the results of this study have implications for business owners and communities living in the affected area. Moreover, findings have revealed practical strategies and interventions that may be used by micro- to medium-scale businesses to mitigate the socioeconomic impact of sewage spillages.

**Contribution:**

The practical strategies revealed in this study contribute to reducing the vulnerability of communities and businesses to sewage spillages. Findings of the study have also been disseminated to the business owners and other relevant stakeholders to provide them with alternative solutions in ‘softening the blow’ caused by the spillages.

## Introduction

Globally, there is a continued focus by several key stakeholders on ensuring that clean and safe water is provided to the world’s population. The Sustainable Development Goal (SDG) 6 focuses on the quality of water resources and providing all people access to clean water and sanitation (Tortajada & Biswas 2018). Water pollution poses a significant challenge that impedes the attainment of SDG 6. Water pollution is caused by many factors, for example, damaged water infrastructure, leakage from water tanks, heavy rain, industrial or domestic waste release, flooding, marine dumping, sewage overflows, or human error (Haseena et al. 2017). Consequently, the issue of water contamination and access to safe drinking water is a global and national concern (World Health Organization [WHO] 2022). Although water pollution has many causes, sewage spillage is one of the leading causes of water pollution (Edokpayi, Odiyo & Durowoju 2017; Galadima et al. 2011).

The common causes of sewage spillage are when water infrastructure and sewage systems get strained because of heavy rain, flooding, and outdated or poorly maintained infrastructure, which results in wastewater being released into rivers and oceans (Hattingh 2022). The impacts of poorly managed wastewater treatment plants (WWTPs) on water quality result in the ineffective pumping and treatment of wastewater (Hallowes 2019). Using old, poorly maintained or damaged WWTPs infrastructure releases sewage and wastewater that pollutes the environment and severely impacts communities and water resources (Edokpayi et al. 2017). This significantly impacts communities in the affected area, including chemical, social, economic, and environmental effects.

Sewage has organic and inorganic compounds, such as heavy metals, pharmaceuticals, drugs, nitrogen and phosphorous compounds, and other contaminants (Arumugam et al. 2018). When sewage infiltrates water sources, it often contaminates these vital resources, posing significant health risks to humans, marine ecosystems, and wildlife. As a result, the contaminated water can lead to health risks to people, marine life, and animals. Consequently, sewage spillage can facilitate the transmission of diseases and environmental pollution (Edokpayi et al. 2017). A study in New Zealand highlights the vulnerability of the ageing wastewater infrastructure to damage from extreme weather conditions, resulting in blockages, overflows, and infrastructure deterioration (Hughes et al. 2021). Moreover, research conducted in Canada and the United States of America has focused on disease outbreaks stemming from water pollution caused by sewage discharge (Hrudey, Hrudey & Pollard 2006). These developments have far-reaching implications for the well-being of local communities.

Sewage spills not only pose environmental and health concerns but also have profound implications for the well-being of communities residing in affected areas (Anderson & Gerber 2007). For example, the foul odours, unpleasant sights of the spills, and health issues can affect the quality of life of communities in these areas. Consequently, individuals may find it challenging to navigate the affected areas and maintain their daily routines, including commuting, shopping, and engaging in leisure activities (Sojobi & Zayed 2022). Furthermore, popular recreational spots such as beaches, restaurants, theme parks, and local parks may be forced to close, limiting community members’ opportunities for leisure and relaxation. These closures can also have a detrimental impact on tourism in the region, exacerbated by negative publicity surrounding sewage spillage. More so, coastal towns that depend on tourism for their economy will be affected because of the reduced number of tourists visiting these areas. This will have a negative impact on the economy of the affected area. The value of properties may also decrease and affect the real estate market (Throupe et al. 2013). Sewage spillage can also cause damage to infrastructure. Thus, because of sewage spillages, there will be repair or replacement costs to community members and businesses in the affected area. This will be an additional economic consequence of sewage spillage. Moreover, sewage-related water pollution fosters the proliferation of algae, leading to eutrophication: a phenomenon characterised by diminished oxygen levels in the water, which harms marine life (Singh & Gupta 2016). As a result, the marine ecosystem will be affected, causing harm to fish, other aquatic organisms, and plants as they rely on a balanced ecosystem and clean water. This can also affect humans who consume contaminated fish.

Sewage spillage is also a concern in South Africa because of the deteriorating infrastructure and substandard service delivery (Mazele & Amoah 2022). This is also true in the KwaZulu-Natal province of South Africa, where safe drinking water is scarce (Thakur et al. 2019). The Durban floods in April 2022 exacerbated the strain on the already fragile water infrastructure (Comins 2023). The aftermath of these floods, characterised by inadequate water infrastructure (Wall 2007) and insufficient maintenance (Rudolf & Odeku 2019; Vhahangwele & Duncker 2016), saw significant damage to sewage pumps at eight treatment plants (Khan 2023). This resulted in sewage from the Onhlanga treatment plant spilling into the Umgeni River (Du Plessis 2022) in Durban in the KwaZulu-Natal province of South Africa. Consequently, beaches along the Durban coastline were closed (McCain 2022). The closure of beaches affected the environment and people working and living in these areas. Beaches were closed because of the high amount of *Escherichia coli* (*E. coli*) in the water (Khan 2023) and the flow of untreated chemical and domestic waste from businesses, households, and industries in the city (Carnie 2022). In addition, repairs to the damaged water infrastructure in the area needed to be undertaken (Papayya 2022). Thus, the sewage spillage negatively impacted the tourism industry in Durban, and the suburbs along the Durban North coastline were the worst affected areas (Du Plessis 2022).

Moreover, the coronavirus disease 2019 (COVID-19) pandemic prompted the South African government to implement a nationwide lockdown in March 2020, which included the closure of all South African borders. The national lockdown significantly impacted cities and towns that relied on tourism to sustain the local economy. Businesses in Durban were trying to recover after the COVID-19 pandemic when the floods in Durban and poorly maintained infrastructure led to sewage spillages and further economic losses. Similarly, research (Bhoola 2022) revealed that COVID-19 caused substantial economic losses to businesses in Durban. Likewise, Umhlanga Rocks is a coastal resort town that depends on tourism and business activities to sustain the local economy (Ramsaru 2011). These losses were even more significant because of beach closures, resulting in businesses along the coastline area closing or having shorter operational hours (Papayya 2022). Although methods to address the issues causing sewage spills take time and are expensive (Pillay, Machete & Hart 2022), efforts have been made to prevent further sewage overflow by using early warning systems and improving flood protection structures (Singh et al. 2022). Hence, addressing the issue of sewage spillages is an essential step to prevent water pollution and protect water resources for both humans and the environment. Therefore, the aim of the study is twofold: (1) to assess the socioeconomic impacts of sewage spillages as experienced by the participating business owners and (2) to determine the association between the socioeconomic impacts of sewage spillages and sociodemographic variables relevant to businesses.

Accordingly, the study intends to provide a critical awareness of these impacts, which can provide new knowledge and practical strategies to support businesses to prepare for and lessen the effects of future sewage spillages. The study intends to empower local business owners and community members to think about the sewage spillage incidents in Durban and its effect on the local economy. Furthermore, studying these impacts of spillages on businesses is broader than economic losses as it has implications for the sustainability of businesses, public health, environmental responsibility, and policy development. The insights gained from this study can, therefore, assist in promoting economic growth, enhance public health, inform policy development, and encourage responsible environmental management.

## Research methods and design

**FIGURE 1:**
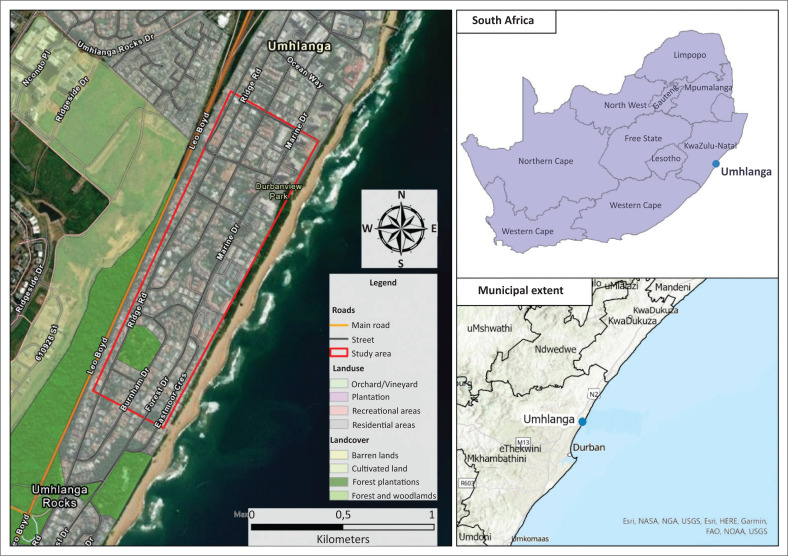
Map of the study area with the research boundary.

This cross-sectional study was conducted to investigate the socioeconomic impacts of sewage spillages on businesses along the Umhlanga Rocks coastline area (Figure 1). This seaside suburb is located north of Durban, in the eThekwini Municipality in the KwaZulu-Natal province of South Africa. The main Umhlanga beach was one of the worst affected beaches in the north of Durban because of the sewage spillages (Du Plessis 2022). Immediately after the flooding in April 2022, all beaches along the Durban coastline were closed (Carnie 2022). Furthermore, in August 2022, 13 beaches were closed because of the high levels of *E. coli* in the water (Singh 2022). The beach closures created public health hazards impacting tourism and other business activities along the affected coastlines (Bouchard 2023; Du Plessis 2022; Kotze 2023; Marriah-Maharajh 2022; Naidoo 2022).

Thus, a scoping phase was conducted whereby the researcher travelled to the main Umhlanga Beach coastline area to view the activities and business operations at the site. The scoping phase assisted in contextualising existing knowledge; for example, businesses that were currently operational were identified, and the extent of the sewage spillages damage was also observed. Consequently, the scoping phase was used to evaluate the study’s nature, extent, and scope (Levac, Colquhoun & O’Brien 2010), and assisted with the study’s inclusion and exclusion criteria (Madanian, Norris & Parry 2020).

The research instrument for this study was a questionnaire. The questionnaire was selected as the best method as it was more accessible and less time-consuming for participants to answer, as it took approximately 10 min to complete. The questionnaire comprised open and closed-ended questions and was answered at a suitable time and place for the participants. With the exception of the sociodemographic variables, the questionnaire sought to investigate four socioeconomic aspects: (1) awareness of the impact, (2) economic impact of sewage spillages, (3) mitigation measures, and (4) early warning and identification of damaged water infrastructure (Table 1). Each aspect was represented by 10 closed-end questions and 1–2 open-ended questions, with the former using a Likert scale to indicate participants choice, that is 1 = disagree, 2 = neutral and 3 = agree.

**TABLE 1:** Aspects of the questionnaire.

Sociodemographic	Awareness of impact	Economic impact	Mitigation measures	Early warning
Age of business	Local economy	Experienced losses	Business loan	Awareness of systems
Business size	Impacts businesses	Damage to business	Government relief fund	Investments in equipment
Business sector	Proximity of business	Damage to equipment	Customers promotion	Efforts made
	Closure of business	Reputational damage	Customers discounts	Awareness of damaged systems
	Disruption of business	Business was closed	Marketing strategies	Maintenance of infrastructure
	Damage business reputation	Operational hours reduced	Advertising strategies	Inspection of infrastructure
	Costly clean-up	Business operations reduced	Negotiated reduced costs from suppliers	Methods to detect infrastructure
	Costly repairs	Paid for clean-up costs	Varied business products	Methods to monitor infrastructure
	Maintenance reduces spillage	Paid for repair costs	Varied business services	Stakeholders’ involvement
	Wider economic impacts	Insurance covered losses	Used other mitigation measures	Technological knowledge

Recruitment for this study was essential for ensuring that an adequate sample size for the target population was selected. It involved identifying participants, communicating with them about the study and obtaining their consent to participate (Nusbaum et al. 2017). The operational businesses in the research area were screened to determine eligibility for this study. Screening to recruit participants assisted in selecting the most appropriate participants for this study (Martinson et al. 2010). Thus, a purposive sampling method was used to recruit participants. Business owners who were sole proprietors were approached, and the nature of the study was explained. Subsequently, their participation was solicited, and participants were provided with an informed consent letter that explained the study and data-collection process. If participants were unwilling to participate in this study, the next business owner in the same area was approached.

### Ethical considerations

Ethical clearance to conduct this study was obtained from the University of KwaZulu-Natal Humanities and Social Sciences Research Ethics Committee (HSSREC) (No. HSSREC/ 00005545/2023). For this study, the participant’s rights, anonymity, and confidentiality were safeguarded, and the researcher confirmed that codes or pseudonyms would be used to protect the identity of the participants.

Prior to the commencement of the data analysis, data entry was conducted, which involved the inputting of data from the completed physical questionnaire into Microsoft Excel. For the qualitative section, the results were analysed thematically. Owing to its flexibility, thematic analysis is used in several disciplines, such as international purchasing in retail firms, higher education, chronic disease management, and understanding stakeholders’ experiences in urban regeneration projects. Although this tool is widely used in qualitative research (e.g. Nzimande 2023), limited attention has been paid to the finer details of thematic analysis, such as what such analysis is and what steps are needed to perform it. Thematic analysis and content analysis are similar in terms of their philosophical foundation, data coding, and finding themes that can explain a phenomenon from different perspectives (Vaismoradi et al. 2016). However, differences between the two methods also exist, including the ways in which content analysis can be performed on non-textual data, such as videos, and how the results are presented in conceptual models. To address these limitations, some scholars have explicitly grounded the different phases of the process of thematic analysis. These phases are related to those of ground theory in that they include the initialisation, construction, rectification, and finalisation phases, each broken into stages (Braun & Clark 2006; Charmaz 2006; Vaismoradi et al. 2016).

Therefore, line-by-line coding of the open-ended questions was conducted manually through the assistance of Excel. This step involved the tagging of text from the participants (verbatim) so that sense could be made of them. There was a total of 897 verbatim texts from the participants yielding nine codes, which were then grouped into subthemes and then three main themes. Notably, some verbatim texts matched more than one theme as the themes were interlinked.

The quantitative data analysis involved a series of statistical tools. Firstly, the Shapiro–Wilk test was utilised to assess the normality of the data distribution. All calculated *p*-values were found to be less than the predetermined significance level (α = 0.05), indicating a departure from normal distribution. This supported the use of non-parametric statistical analysis methods for comparing multiple groups of businesses.

To gauge the socioeconomic impacts of sewage spillages as perceived by business owners, means were computed to determine the average responses for each dependent variable. Additionally, standard deviations were calculated to gauge the spread of responses around the means. Furthermore, to investigate the relationship between business ownership and the considered dependent variables concerning the economic impacts of sewage spillages, the Pearson chi-square test was employed at a 5% significance level.

Box plots were employed for graphical representation to assess central tendencies, variabilities, and potential outliers across different sociodemographic categories to explore the connection between the socioeconomic consequences of sewage spillages and various business sociodemographic factors. The Kruskal–Wallis test was then applied to ascertain whether these sociodemographic variables significantly influenced the socioeconomic impacts of sewage spillages on businesses. Subsequently, a significant Kruskal–Wallis test was followed by Dunn’s test to pinpoint specific differences between groups within the sociodemographic variables in terms of their responses to various factors linked to the socioeconomic impacts of sewage spillages on businesses. Finally, the practical significance of differences was quantified through the Cohen’s d test for those sociodemographic groups displaying significant differences in their responses to different factors.

## Results

### Quantitative results

The analysis of mean ratings across different aspects of the socioeconomic impacts of sewage spillages on businesses revealed noteworthy trends. Notably, the highest mean ratings were observed within the economic impacts aspect, while the lowest mean ratings were associated with early warning systems. This suggests that business owners placed higher importance on the financial repercussions of sewage spillages, while the early warning systems were rated lower in terms of significance. To further elucidate, the standard deviations in the overall responses for each factor related to these aspects indicated relatively low variability for mitigation efforts and early warning system aspects. This is evidenced by their proximity to the corresponding means in Table 2, signifying a higher level of consensus among respondents for these particular aspects.

**TABLE 2:** Means, standard deviations and *p*-values for the overall perceptions of economic impacts of sewage spillages by business owners.

Factors	Mean	Standard deviations	Chi-square (*p*-value)
**Awareness factors**			
Negatively impacts economy	2.82	0.50	0.30
Negatively impacts business	2.85	0.46	0.02**
Location affects loss	2.74	0.59	0.54
Closure of business	2.62	0.73	0.30
Disruption of business	2.75	0.64	0.01**
Customer loyalty and trust	2.77	0.55	0.06
Clean-up is expensive	2.75	0.53	0.48
Cost of repairs is expensive	2.79	0.52	0.58
Regular maintenance lessens economic impact	2.80	0.54	0.44
Economic effect can spread to the wider community	2.85	0.48	0.68
**Economic impact factors**			
Business experienced losses	2.78	0.55	0.16
Damage to business	2.23	0.92	0.99
Damage to business equipment	1.79	0.87	0.70
Reputational damage	2.35	0.87	0.80
Business closed	1.87	0.93	0.48
Operational hours reduced	2.63	0.74	0.04**
Day-day operations was affected	2.66	0.69	0.52
Paid for clean-up costs	2.00	0.95	0.25
Paid for repair costs	1.93	0.95	0.64
Insurance covered losses	1.83	0.92	0.38
**Mitigation measures factors**			
Applied for a business loan	2.34	0.09	0.82
Applied for relief funds	1.79	0.08	0.93
Offered promotions	2.57	0.08	0.46
Offered discounts	2.60	0.07	0.03**
Changed marketing strategy	2.68	0.07	0.88
Changed advertising strategy	2.69	0.06	0.14
Reduced prices with suppliers	2.32	0.08	0.16
Varied business products	2.08	0.09	0.66
Varied business services	2.06	0.08	0.90
Used other mitigation measures	2.36	0.08	0.13
**Early warning and identification of damaged water infrastructure factors**			
Awareness of early warning systems	1.28	0.06	0.12
Investment in equipment	1.23	0.06	0.17
Efforts for developing methods for early warning systems	1.24	0.05	0.27
Awareness of damaged infrastructure	1.24	0.06	0.27
Maintenance of infrastructure	1.28	0.06	0.03**
Inspection of water infrastructure	1.31	0.06	0.04**
Methods used to detect damaged infrastructure	1.29	0.06	0.45
Methods used to monitor damaged infrastructure	1.34	0.06	0.57
Discussions between business owners and other stakeholders	1.31	0.06	0.07
Knowledge of technology to improve the identification of water infrastructure damage	1.52	0.06	0.24

** , Significance level < 0.05.

The chi-squared test yielded *p*-values less than 0.05, signifying statistically significant associations between business owners’ perceptions and certain factors within each aspect of the study, including economic impacts, business strategies, and early warning systems. Specifically, within the economic impact’s category, statistically significant ratings were observed for factors such as awareness of the negative impacts of sewage spillages on business (*p*-value = 0.02), awareness of business disruptions (*p*-value = 0.01), and reduction in operational hours (*p*-value = 0.04), as outlined in Table 2. Regarding mitigation measures, offering discounts emerged as the sole overall significant mitigation measure employed by business owners (*p*-value = 0.03), while within the early warning systems aspect, the maintenance of infrastructure (*p*-value = 0.03) and inspection of water infrastructure (*p*-value = 0.04) were identified as the only overall significant factors, as indicated in Table 2.

**FIGURE 2:**
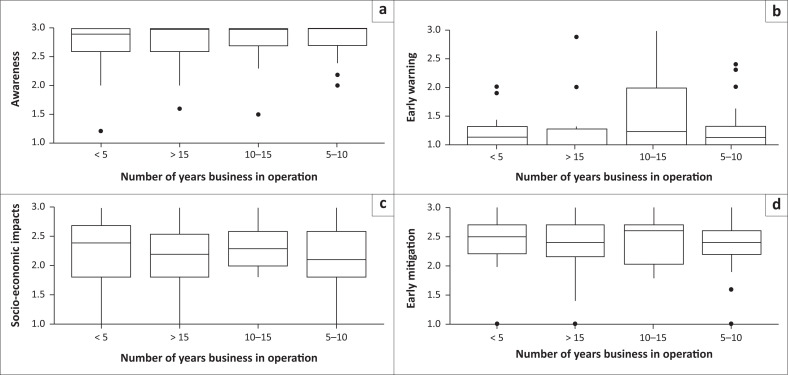
Boxplots of average responses to the different aspects; (a) awareness, (b) early warning, (c) socioeconomic impacts, (d) mitigation measures; by number of years in business operation.

**FIGURE 3:**
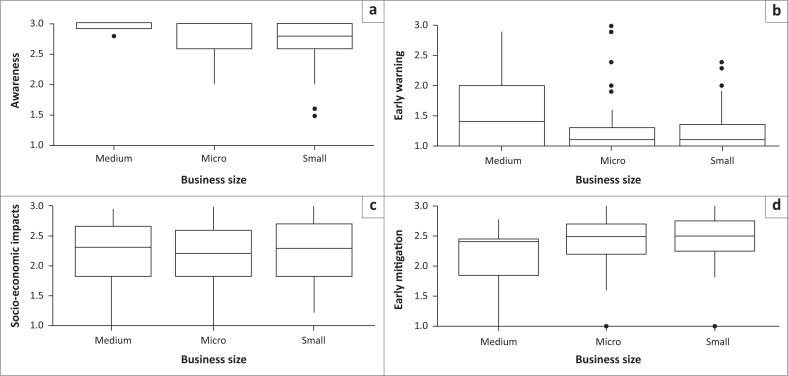
Boxplots of average responses to the different aspects; (a) awareness, (b) early warning, (c) socioeconomic impacts, (d) mitigation measures; by business size.

**FIGURE 4:**
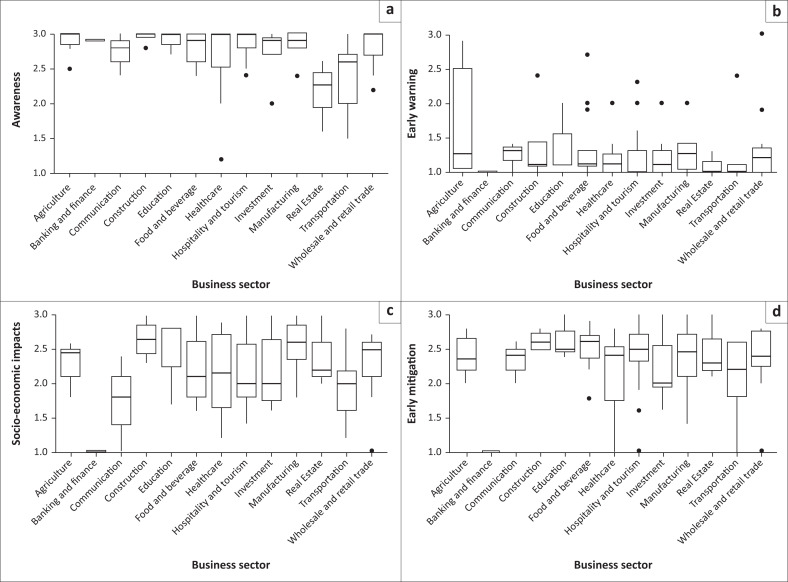
Boxplots of average responses to the different aspects; (a) awareness, (b) early warning, (c) socioeconomic impacts, (d) mitigation measures; by business sector.

Boxplots display the distribution of average responses to the different aspects (awareness, socioeconomic impacts, mitigation measures, and early warning) related to different categories of business sociodemographic variables (Figure 2, Figure 3 and Figure 4). The median (line inside the box), quartiles (box edges), and any potential outliers (dots) are presented. Results provided visual indications of differences in the average responses (medians and quartile ranges) to the different aspects related to the socioeconomic impacts of sewage spillages on businesses.

The Kruskal–Wallis test showed that significant associations exist between the different sociodemographic variables and the aspects of socioeconomic impacts of sewage spillages considered in this study (Table 3). Notably, when assessing responses from business owners across different business sectors, it was observed that each factor related to the socioeconomic impacts received identical ratings within these sectors. This lack of variability precluded the application of the Kruskal–Wallis test for comparisons between business sectors. Significant associations were shown to exist between different factors within the various aspects of socioeconomic impacts of sewage spillages and the business sizes, while no statistically significant associations were made with the number of years that businesses were in operation at a *p*-value < 0.05 (Table 3). Factors that showed to be significantly associated with business size included the awareness of the socioeconomic impacts of sewage spillages on customer loyalty and trust (*p*-value = 0.01) and early warning factors of maintenance of infrastructure (*p*-value = 0.01), inspection of water infrastructure (*p*-value = 0.01), and the discussions between business owners and other stakeholders (*p*-value = 0.02).

**TABLE 3:** Kruskal–Wallis test of significance for different sociodemographic variables.

Factors	Number of years business in operation			Business size		
	Chi-squared statistic	*df*	*p*-value	Chi-squared statistic	*df*	*p*-value
**Awareness factors**						
Negatively impacts economy	4.41	3	0.22	3.33	2	0.190
Negatively impacts business	5.45	3	0.14	5.54	2	0.060
Location affects loss	1.23	3	0.75	1.57	2	0.450
Closure of business	0.83	3	0.84	1.32	2	0.520
Disruption of business	1.79	3	0.62	4.45	2	0.110
Customer loyalty and trust	2.64	3	0.45	8.57	2	0.014**
Clean-up is expensive	2.69	3	0.44	3.13	2	0.210
Cost of repairs is expensive	2.03	3	0.56	2.10	2	0.350
Regular maintenance lessens economic impact	1.29	3	0.73	3.69	2	0.150
Economic effect can spread to the wider community	0.61	3	0.89	2.11	2	0.350
**Economic impact factors**						
Business experienced losses	0.70	3	0.87	3.40	2	0.180
Damage to business	2.44	3	0.49	0.23	2	0.890
Damage to business equipment	1.97	3	0.58	1.54	2	0.460
Reputational damage	1.08	3	0.78	2.45	2	0.290
Business closed	0.75	3	0.86	0.36	2	0.830
Operational hours reduced	2.52	3	0.47	3.50	2	0.180
Day-day operations was affected	0.78	3	0.85	0.02	2	0.980
Paid for clean-up costs	1.34	3	0.72	2.45	2	0.290
Paid for repair costs	1.85	3	0.60	0.08	2	0.960
Insurance covered losses	3.08	3	0.38	3.77	2	0.150
**Mitigation measures factors**						
Applied for a business loan	0.10	3	0.99	0.22	2	0.900
Applied for relief funds	0.76	3	0.86	0.19	2	0.910
Offered promotions	4.44	3	0.26	2.09	2	0.350
Offered discounts	3.99	3	0.27	5.27	2	0.070
Changed marketing strategy	2.33	3	0.51	1.06	2	0.590
Changed advertising strategy	1.26	3	0.74	4.24	2	0.120
Reduced prices with suppliers	1.43	3	0.70	4.91	2	0.090
Varied business products	0.61	3	0.89	1.92	2	0.380
Varied business services	3.89	3	0.27	0.91	2	0.630
Used other mitigation measures	0.92	3	0.82	1.87	2	0.390
**Early warning and identification of damaged water infrastructure factors**						
Awareness of early warning systems	2.74	3	0.43	3.98	2	0.140
Investment in equipment	0.76	3	0.86	5.08	2	0.080
Efforts for developing methods for early warning systems	1.67	3	0.64	4.67	2	0.100
Awareness of damaged infrastructure	5.45	3	0.14	3.18	2	0.200
Maintenance of infrastructure	2.88	3	0.41	9.92	2	0.010**
Inspection of water infrastructure	4.03	3	0.23	8.84	2	0.010**
Methods used to detect damaged infrastructure	6.97	3	0.07	2.75	2	0.250
Methods used to monitor damaged infrastructure	7.00	3	0.07	1.55	2	0.460
Discussions between business owners and other stakeholders	2.31	3	0.51	8.20	2	0.020**
Knowledge of technology to improve the identification of water infrastructure damage	4.27	3	0.23	4.87	2	0.090

** , Significance level < 0.05.

The Dunn’s test was used to assess the significance of differences in responses between paired groups of business sizes (micro, small, and medium) in terms of their responses to the factors related to the socioeconomic impacts of sewage spillage (Table 4). Results highlighted that medium-sized businesses consistently showed different responses compared to micro and small businesses across various aspects. In terms of ‘customer loyalty and trust’, medium-sized businesses differed significantly from both micro and small businesses, with *p*-values of 0.01 and 0.03, respectively. A similar trend was observed in the ‘maintenance of infrastructure’ aspect, where medium-sized businesses significantly differed from micro and small businesses, with *p*-values of 0.00 and 0.03.

**TABLE 4:** Dunn’s test multiple comparisons with *p*-values adjusted with the Bonferroni method.

Group	Z	*p*.unadjusted	*p*.adjusted
Customer loyalty and trust			
Medium-Micro	2.85	0.00	0.01**
Medium-Small	2.39	0.02	0.03**
Micro-Small	−0.38	0.71	0.71
**Maintenance of infrastructure**			
Medium-Micro	3.15	0.00	0.00**
Medium-Small	2.44	0.01	0.03**
Micro-Small	−0.71	0.48	0.48
**Inspection of water infrastructure**			
Medium-Micro	2.96	0.00	0.01**
Medium-Small	2.16	0.03	0.06
Micro-Small	−0.89	0.38	0.38
**Discussions between business owners and other stakeholders**			
Medium-Micro	2.85	0.00	0.01**
Medium-Small	2.39	0.02	0.03**
Micro-Small	−0.38	0.71	0.71

** , Significance level < 0.05.

Moreover, the ‘inspection of water infrastructure’ and ‘discussions between business owners and other stakeholders’ aspects showed the same pattern. In the former, the medium-micro comparison showed a significant difference (*p*-value of 0.01), and in the latter, both medium-micro and medium-small comparisons showed significant differences with *p*-values of 0.01 and 0.03, respectively. To further assess the practical significance of these differences, the z-scores corresponding to the significant *p*-values (< 0.05) from the Dunn’s test were calculated. These z-scores support that the differences between the various groups of business sizes concerning the aspects related to the socioeconomic impacts of sewage spillages are statistically significant.

The Cohen’s d test was used to assess the practical significance of the differences found between the different business sizes in the Dunn’s test. In terms of ‘Customer Loyalty and Trust’, the large effect size of 7.06 (Table 5) indicated that the medium-sized businesses differ significantly from their smaller counterparts and the difference holds significant practical impacts. In terms of infrastructure-related factors, both ‘Maintenance of Infrastructure’ and ‘Inspection of Water Infrastructure’ showed major effect sizes of 3.16 and 3.14, respectively. This indicates that medium-sized businesses, as identified by the Dunn’s test, differ in their approaches to maintaining and inspecting infrastructure compared to Micro and Small businesses and have significant practical impacts. Lastly, in terms of ‘Discussions between Business Owners and other Stakeholders’, the effect size of 3.16 reinforces the Dunn’s test findings, indicating that medium-sized businesses significantly differ from their smaller counterparts in engaging with stakeholders in a practically meaningful manner.

**TABLE 5:** Cohen’s d effect size.

Variable	d estimate	CI-upper	CI-lower	Effect
**Awareness factors**				
Customer loyalty and trust	7.06	6.29	7.82	Large
**Early warning and identification of damaged water infrastructure factors**				
Maintenance of infrastructure	3.16	2.73	3.59	Large
Inspection of water infrastructure	3.14	2.72	3.57	Large
Discussions between business owners and other stakeholders	3.16	2.73	3.59	Large

CI, confidence interval.

### Qualitative results

Nine questions from the survey were open-ended and thus provided participants with opportunities to elaborate on all the main aspects of the study. Three main themes emerged from the dataset: economic impacts, business strategies, and early warning systems. These themes are presented and discussed with the assistance of unedited verbatim data.

#### Theme 1: Economic impacts

The first theme is centred on the economic impacts of sewage spillages on businesses located alongside Umhlanga beach. Business owners asserted that the 2022 spillages adversely affected their businesses, with little to no business spared. The majority of the participants shared their grievances about how their businesses lost capital because of a lack of sales during the closure of businesses, which had a domino effect on laying staff:

‘I know businesses that closed due to what they experienced and lost a lot of foot traffic. The area, the smell, the aftereffects impact the community and steer them away.’ (P1)

‘I work in the food industry. I could not sell all my products. I lost money from sales and my products went bad.’ (P52)

Because of the unpredictability of the spillages, businesses could not inform their customers when was the right to or the wrong time to come and shop, which caused issues where businesses would call in the staff for work, restock supplies and then have to unexpectantly close because of the spillages. The following participants said:

‘My operational hours were affected and I lost sales at my pharmacy. Many loyal customers have now gone to other pharmacies, this will have a long-term effect on my business.’ (P17)

‘I lost income and sales. Had fewer customers. My operations were disrupted. I let customers down and I lost this business sales.’ (P49)

Furthermore, though the current study focused on the 2022 incidents, the eThekwini Municipality is still experiencing sewage spillages, causing further economic burdens on business owners. Therefore, several participants raised concerns as the current spillages further exacerbate the 2022 spillages, giving business owners no time to recuperate from their losses:

‘… Reputation has been tarnished.’ (P14)

‘Yes. I am still trying to recover. Especially since the sewage spill is ongoing.’ (P102)

#### Theme 2: Business strategies

To share possible and helpful solutions with other businesses facing similar issues, the participants were requested to include the strategies that helped (and possibly still helping them) during the sewage spillages. To prevent businesses from permanently closing down, numerous businesses mentioned receiving assistance from financial institutions, while others could claim from their insurance brokers.

‘Applied for bank loans and took out insurances … very effective paid rent and resolved some repairs required.’ (P4)

Others made use of innovative and different marketing strategies to attract customers:

‘I offered online specials. I offered free delivery of products. I reduced prices … It brought in some income but not enough.’ (P39)

‘Had various discounts and promotions. Changed my business models … It was effective. I achieved some extra business.’ (P1)

These narratives indicate that business owners in the area were constantly revolving and changing their marketing and advertising strategies to ensure they remained operational. These mentioned practices indeed worked, as the businesses have been operational for more than a year as the sewage spillages. However, looking at the costly experiences of some of these businesses, it is clear that the lessons learnt here were enough to warn other businesses from operating in a similar area, amongst others:

‘Move further away from sewage do not locate business close by. Depending on nature of business avoid going into areas by affected sewage spillage.’ (P3)

‘Become proactive in finding solutions and implementing the solutions. Hold authorities to account for old or collapsed infrastructure, maintenance and repair and quality of services. (P8)

‘Choose business premises wisely. Be aware of the surroundings. Develop alternative ways of raising income from the same business. For example, use free deliveries.’ (P103)

#### Theme 3: Early warning

Thinking proactively and with the aim of assisting business owners in identifying damaged water infrastructure and thus assisting the municipality in providing early warnings of the spillages, participants were requested to share ideas on what critical indicators of water infrastructure damage should the Municipality and other relevant stakeholders be monitoring. Unsurprisingly, the issue of the lack of maintenance of water pipes was glaring:

‘Get engineers to test the infrastructure on an annual basis. Fix whatever breaks no matter how small it is.’ (P11)

‘Monitoring pH levels regularly.’ (P80)

‘Cracks and leaks. Soil and plants around the damaged water infrastructure are swamped or have excess water. Fish float ashore or lie face up showing there is something wrong.’ (P58)

With the ever-increasing number of the world’s population migrating to urban areas, human waste is also increasing. Therefore, urban planners, municipal employees, engineers, and other urban stakeholders are constantly finding ways to use technology to help detect and alert communities of possible infrastructure damage before it becomes a significant problem. In the same breath, participants were asked to suggest possible technological strategies that could assist in early detection:

‘The use of telecoms to monitor. Measuring and monitoring devices will give real time indications of problems. Telemetric systems.’ (P8)

‘Detectors and sensors can check and alert us when they are leaks and cracks. Using mobile devices we can be alerted by different people about real time information and this can be used to monitor the affected areas.’ (P49)

‘Monitor and evaluate water quality. Cameras can give high resolution images of areas to show changes in vegetation or soil.’(P58)

However, the use of these technological tools is expensive and in a poverty-ridden country such as South Africa, how sustainable are these technologies is a question that one participant alluded to:

‘At what cost? I don’t think our country is ready for this type of devices.’ (P6)

In summary, the given comments from the business owners operating in the Umhlanga beachside show their concerns with the lack of long-term solutions to the sewage spillages issues that threaten their livelihood. And although there have been government relief funds and other financial avenues available to assist companies in response to further risks and damage caused by the spillages, these do not seem to be sustainable as there are only so many business loans and reliefs that the business owners could apply for and possibly pay back.

## Discussion

The purpose of the study was twofold: (1) to assess the socioeconomic impacts of sewage spillages as experienced by the business owners, and (2) to determine the association between the socioeconomic impacts of sewage spillages and business sociodemographic variables. Firstly, results suggest that the sewage spillages drastically disturbed the business by reducing operational hours; to combat this capital loss, discounts were offered to customers; and to improve the early warning systems in place, participants suggested maintenance of these water infrastructures. Secondly, all of the business sizes perceived the sewage spillages differently.

The current study provides empirical evidence on the different socioeconomic aspects of sewage spillages and how they affect business owners. Thus, it is one of two studies that investigated this research problem in South Africa. Furthermore, the strengths of the study lie in its large sample size, thereby increasing the precision of the results. However, the study did have some limitations. Firstly, as this was a cross-sectional study and while statistical significance can provide insights, it may not necessarily imply causal relationships. Secondly, study findings may not be generalisable to other coastal cities facing similar issues. Furthermore, the analysis focuses on the provided categorical variables, and additional unexplored factors may contribute to the results attained. Despite this, the results of this study do support prior studies and may be used to introduce further interventions to assist small-to-medium scale businesses in reducing the effects of sewage spillages.

Firstly, the results observed several socioeconomic impacts of the sewage spillages experienced by the businesses. Wastewater systems’ purpose is to transport the waste produced in homes, businesses, and hospitals away from the source; however, when these treatment plants exceed their capacity or are poorly maintained, WWTPs become ineffective (Hallowes 2019). In the eThekwini Municipality, the 2022 floods caused severe damage not only through the loss of lives (Hattingh 2022) but also through sewerage infrastructure damage caused by blocked or broken sewerage pipes (Magubane 2022). Similarly, other disasters are said to have caused extensive damage to sewerage systems, such as the Great East Japan Earthquake (Matsuhashi et al. 2014) and landslides (Vranken et al. 2013). Results indicated that sewage spillages have immense consequences on businesses by negatively affecting and disrupting the flow of business, as evidenced in the study. Particularly, micro, medium, and small-scale businesses are greatly affected as these spills significantly damage their reputation as the putrid stench from the spills would mean closing down the business by reducing operational hours, thus losing customers. These results corroborate similar studies that have found that spills reduce productivity (Sindane & Modley 2022) and increase unemployment (Adam 2022).

Beach closures have a rippling effect on businesses as fewer tourists on the beachfront mean less traffic and thus fewer customers, which causes businesses to close down or drastically reduce their profits. For the 2022/2023 Blue Flag Programme, which is run by the Foundation for Environmental Education, the eThekwini Municipality lost the Blue Flag status of beaches because of the ever-increasing levels of *E. coli* which caused several beaches to be closed off to the public. Besides, *E. coli*, lab results also showed high levels of faecal streptococcus, which may be attested to the April 2022 floods and ageing sewerage infrastructure. This simply meant that fewer customers were visiting businesses that caused an approximate R25 million a day to be lost by the accommodation and hospitality sector within the Umhlanga area (Phooko 2022). Thus, it is evidence that the deterioration of the water quality has detrimental impacts on the businesses. These effects are not only limited to the businesses, but media reports by Groundup have argued that property owners have complained about how sewage spills reduce their property values (van Schalkwyk & Sofianos 2020).

Secondly, the results offered insights into the association between socioeconomic impacts and business sizes. Medium businesses, compared to micro and small businesses, largely agree that sewage spillages significantly influenced customer loyalty, trust, and business reputation. It is interesting that micro-scale businesses did not find these factors to be influenced by the spillages, which contradicts Dacko-Pikiewicz (2022) but agrees with Potocki and Wierzbinski (2014), who found that having a good reputation is a strategic intangible asset for building trust and customer loyalty that is used by new, micro-scale businesses. Furthermore, when looking at the early warning and identification of damaged water infrastructure, medium-scale businesses were neither satisfied nor dissatisfied with infrastructure maintenance compared to their counterparts. In fact, the micro and small business owners were dissatisfied with the infrastructure maintenance and the discussions between the owners and other stakeholders.

### Implication of study

The results of this study have implications for government, policymakers, community members, and business owners. There is a need for updating current regulatory measures to ensure accountability for preventing sewage spillages, improvement of and investment in resilient sewage infrastructure by government, community engagement and collaboration by encouraging community outreach programmes to increase awareness, reporting and monitoring of sewage spillage incidents, and the development of emergency response plans and risk assessments by business owners. The practical strategies revealed in this study have implications for business owners in reducing their vulnerability to sewage spillages. Accordingly, the study has implications for business owners when planning for the future by considering the long-term effects of sewage spillage incidents.

Finally, the study has implications and recommendations for strategic development. Existing municipality strategies need to be reviewed to include improved response strategies, enhanced regulatory measures to prevent future sewage spillages, improved sewage infrastructure maintenance monitoring, and effective support mechanisms for all affected businesses. This is particularly important as in the last 3 years, the eThekwini Municipality has been issued with approximately 15 compliance directives. Thus, this study has implications to help communities, business owners, government, and policymakers better understand the risks linked to sewage spillages. Consequently, the repercussions necessitate a comprehensive approach that includes education, raising awareness, practical strategies, investment, and enhanced regulation for mitigating the socioeconomic impacts of sewage spillages.

## Conclusions

This study investigated the socioeconomic effects of sewage spillages on businesses in the Umhlanga Rocks coastline area. Important findings have emerged through the qualitative and quantitative analysis of the data collected. Firstly, this study has demonstrated that sewage spillages did have socioeconomic implications for the affected businesses. Businesses experienced direct and indirect costs, which affected their activities. The direct costs associated with repairs, loss of sales, changing of operational hours, and diversifying marketing and advertising strategies significantly impacted the participants in this study. The findings indicated that business owners had to apply for business loans to repair and replace equipment and infrastructure because of the sewage spills. Moreover, because of the loss of sales, business owners needed to apply for loans to pay their rent and suppliers. In addition, the indirect costs linked with reputational damage, loss of customer loyalty, and trust were also significant. These findings indicated that business owners were concerned about losing their reputation, their customer trust, and loyalty as the indirect costs associated with this loss will continue long after the sewage spillages.

Secondly, the study has also revealed the relationship between sewage spillages and its impact on tourism, which this area relies on. The participants indicated that they had fewer sales and lost income because of less tourists visiting their businesses as a result of the sewage spillage. Tourists were not visiting the affected area because of beach closures and the health hazards linked to the sewage spills. Thus, the findings of this study indicate that tourism decreased significantly in this area. Thirdly, the results of this study offer practical strategies to mitigate the socioeconomic impacts of sewage spillages. These practical strategies include diversifying income sources, maintaining water infrastructure regularly, considering different marketing and advertising strategies and offering discounts to customers. Businesses can proactively safeguard their operations and communities against future sewage spillage incidents using these practical strategies.

Thus, the study provided a comprehensive understanding of the socioeconomic effects of sewage spillages on businesses in the affected area. The results of this study call for an increase in public awareness and education concerning sewage management and the prevention of future sewage spillages. The findings suggest that if communities are more aware of the necessary steps to follow when they first observe sewage spills, then these spills can be managed quickly and effectively. Thus, this awareness and education will help prevent large-scale sewage spills and related damages. Accordingly, the study revealed the need for collaborative efforts among businesses, environmental organisations, government, and communities to mitigate the effects of sewage spillages on the local economy and environment. Through these collaborative efforts, the valuable coastal ecosystems and the businesses that depend on them can be protected for future sustainability.
